# Relationship between air pollution exposure and insulin resistance in Chinese middle-aged and older populations: evidence from Chinese cohort

**DOI:** 10.3389/fpubh.2025.1551851

**Published:** 2025-04-02

**Authors:** Ping Liu, Zhaoliang Zhang, MingZhong Luo

**Affiliations:** ^1^The Second People’s Hospital of Shanxi Province, Taiyuan, Shanxi, China; ^2^The Affiliated YiXing Hospital of Jiangsu University, Yixing, Jiangsu, China

**Keywords:** insulin resistance, triglyceride glucose-related indicators, METS-IR, air pollution, mixture effect

## Abstract

**Aims:**

This study aimed to determine the relationships between mixed exposure to six air pollutants, namely, particulate matter with an aerodynamic diameter of 2.5 micrometers or less (PM_2.5_), PM with an aerodynamic diameter of 10 micrometers or less (PM_10_), sulfur dioxide (SO_2_), nitrogen dioxide (NO_2_), cobalt (CO) and ozone (O_3_), and insulin resistance (IR) indices in Chinese middle-aged and older populations.

**Methods:**

A total of 2,219 participants from the China Health and Retirement Longitudinal Study (CHARLS), who are followed from 2011 to 2015, were included. Surface air pollutant concentration data were obtained from the China High Air Pollutants (CHAP) database. Multivariable linear regression analysis was used to examine the longitudinal associations between different air pollutants and various IR indices. Additionally, Bayesian kernel machine regression (BKMR), weighted quantile sum (WQS) regression, and quantile-based g computation (Qgcomp) were further utilized to assess the mixed effects of the six air pollutants.

**Results:**

Fully adjusted linear models revealed that increases in the levels of the six air pollutants (in μg/m^3^) were associated with higher triglyceride–glucose–body mass index (TyG-BMI; Beta = 0.027–0.128), triglyceride–glucose–waist circumference (TyG-WC; Beta = 0.155–0.674), and metabolic score for insulin resistance (METS-IR; Beta = 0.001–0.029) values during the four-year follow-up period. Further mixture analysis indicated that combined exposure to the six air pollutants had a significant cumulative effect on the increases in these three IR indices. Among the pollutants, NO_2_ and O_3_ were identified as the primary contributor to the cumulative effect. The result of mediation analysis supported the mediating role of BMI in the relationship between air pollution and IR (mediation proportion: 49.1%–93.5%). The results from both subgroup analysis and sensitivity analysis supported the detrimental effects of air pollution on IR.

**Conclusion:**

Both individual and mixed exposures to air pollution were significantly associated with IR in Chinese middle-aged and older individuals, with our study providing new evidence.

## Background

IR is one of the hallmark of diabetes and prediabetes, and is characterized primarily by the body’s biological response to insulin levels being lower than normal (1). IR is one of the most common metabolic disorders worldwide and is associated with various diseases, including type-2 diabetes, hypertension, stroke, and nonalcoholic fatty liver disease (2). Due to global aging and unhealthy lifestyle habits, the prevalence of IR is continuously increasing, contributing to the growing burden of chronic diseases worldwide (3). Focusing on the risk factors for IR is crucial for alleviating the associated disease burden and improving quality of life in populations.

Among various environmental and lifestyle factors contributing to IR, air pollution has emerged as a significant concern. Air pollution is a critical global challenge, severely impacting both the environment and human health. Long-term exposure to air pollution has been reported to affect endothelial function, alter the gut microbiota, the trigger systemic inflammation and IR (3–5). A systematic review identified particulate matter (PM) and nitrogen oxides (NO_x_) as two pollutants that are strongly associated with type-2 diabetes and other metabolic disorders (6–9). Several experiments have shown that exposure to those two air pollutants induces oxidative stress and disrupts insulin signaling pathways in animal models, supporting the metabolic impact of air pollution (10). Meanwhile, body mass index (BMI), a key measure of obesity, has been shown to be a strong mediator between environmental exposure and metabolic disorders. Obesity-related inflammation, insulin signaling disruption, and altered lipid metabolism contribute to the development of IR, and BMI may mediate the effects of air pollution by influencing fat distribution and metabolic function (11, 12). Pollutants such as PM and NO_x_ have been shown to exacerbate obesity-related inflammation by affecting adipose tissue and fat distribution (13, 14). Thus, we hypothesize that BMI may be one of the important mediators of the effect of air pollution exposure on IR. Explaining the mediating role that BMI plays in air pollution’s interference with insulin metabolic pathways would be crucial for developing public health strategies that target environmental risk factors.

Besides, many previous studies in related field have used single-pollutant models, which may not accurately capture the real-world exposure scenarios of individuals who are typically exposed to multiple air pollutants simultaneously. As such, multipollutant models are critical for understanding the complex interactions between different pollutants and their effects on human health (15, 16). In addition, sulfur dioxide (SO_2_), nitrogen dioxide (NO_2_) and ozone (O_3_) are considered as other common air pollutants that significantly impact the health of middle-aged and older individuals (17, 18). Air pollutants such as SO₂, NO₂, and O₃ not only harm the respiratory system but also negatively impact cardiovascular and metabolic health. In addition to its role in metabolic dysfunction, SO₂ exposure has been linked to severe health outcomes, including increased risk of mortality (19). Long-term exposure to NO₂ has been shown to exacerbate adverse outcomes related to respiratory and cardiovascular diseases (20). O₃, a potent oxidant, is associated with an increased risk of mortality from respiratory and cardiovascular diseases, with both short-term and long-term exposure contributing to these outcomes (21). Although experimental evidence of the harmful effects of O_3_ and NO2 is more limited compared to PM, these pollutants are still considered critical indicators of air quality. Given the limited research, further studies are needed to explore which components of air pollution are most strongly associated with IR. Several indices, such as triglycerides and glucose (TyG) index and the metabolic score for insulin resistance (METS-IR), have been developed to assess IR (22, 23). These indices are based on routine biochemical markers and are reliable tools for evaluating IR, closely correlating with the development and outcomes of various cardiovascular and endocrine diseases (24, 25). We intend to use different TyG indices and METS-IR as alternative outcomes to assess IR and conduct a systematic review of the impact of air pollution exposure on IR.

Given the growing body of evidence linking air pollution to IR, this study was designed to test the following specific hypotheses: (1) Exposure to higher levels of air pollutants, particularly PM, NO_2_ and O_3_, is associated with higher levels of IR in middle-aged and older individuals. (2) BMI mediates the association between air pollution and IR, amplifying its adverse metabolic effects.

## Methods

### Study population

The study population was drawn from the first wave (2011) and third wave (2015) of the China Health and Retirement Longitudinal Study (CHARLS), with relevant data collected from each participant during both waves (26). The exclusion criteria were as follows: (1) individuals aged <45 years; (2) individuals with missing data related to the primary outcome variables or covariates; and (3). variables with outliers (any continuous variable that differed from the overall mean by more than 3 standard deviations). Ultimately, this analysis included 2,219 participants (Supplementary Figure 1). Further details about CHARLS can be found in the original study. The CHARLS was approved by the Institutional Review Board of Peking University, and all participants were informed about the disclosure statement and signed informed consent forms (25, 26).

### Assessment of the air pollution chemical composition

The China High Air Pollutants (CHAP) dataset was obtained from the National Earth System Science Data Center.^1^ This dataset provides long-term, full-coverage, high-resolution, and high-quality monitoring data and calculations of surface air pollutants across China. The dataset fully accounts for the spatiotemporal heterogeneity of air pollutants, utilizing artificial intelligence to generate data from big data sources such as ground measurement sources, satellite remote sensing products, atmospheric reanalysis, and model simulations. The dataset has undergone high-quality cross-validation (27–30). The spatial resolution for SO_2_, CO, and NO_2_ is 10 km, whereas that for PM_2.5_, PM_10_, and O_3_ is 1 km. Additionally, we obtained the average Air Quality Index (AQI) for the corresponding prefecture-level cities during the follow-up period to assess the air quality in the participants’ locations. We calculated the average air pollutant concentrations during the follow-up period as the exposure and matched them to the city-level geocodes corresponding to each participant’s residential address (for privacy reasons, it was not feasible to obtain the details of an individual’s residential address from the CHARLS).

### Assessment of IR

Four indices were used as proxies for IR: the TyG, TyG-body mass index (TyG-BMI) TyG-waist circumference (TyG-WC) and metabolic score for insulin resistance (METS-IR). The TyG was calculated as follows: ln [fasting triglyceride level (mg/dL) × fasting glucose level (mg/dL)/2] (31). The METS-IR was calculated as follows: Ln((2 × fasting glucose level (mg/dL) + fasting triglyceride level) × BMI)/Ln (high-density lipoprotein cholesterol level) (32). The calculation formulas for the remaining indices were as follows: TyG-WC = TyG × WC; and TyG-BMI = TyG × BMI. These indices were derived from laboratory tests conducted at both baseline and the end of follow-up.

### Covariates

Covariates were selected from confounding factors identified in relevant studies. The sociodemographic information included sex, age, education level, marital status, place of residence, annual average expenditure, and health insurance status. The lifestyle information included smoking status, alcohol consumption status, the presence of hypertension, diabetes, dyslipidemia, nighttime sleep duration, the use of clean fuel for cooking (to assess indoor air pollution), and daily activity scores (see Supplementary Table 1 for the original scoring table). The blood biomarkers included C-reactive protein (CRP) levels and blood urea nitrogen (BUN) levels. Hypertension was defined as a systolic blood pressure ≥ 140 mmHg or diastolic blood pressure ≥ 90 mmHg, or a self-reported history of hypertension. Diabetes was defined as fasting blood glucose level ≥ 7.0 mmol/L or a self-reported history of diabetes. Dyslipidemia was defined as total cholesterol (TC) level ≥ 240 mg/dL, triglycerides level ≥ 150 mg/dL, low density lipoprotein cholesterol (LDL-C) level ≥ 160 mg/dL, high density lipoprotein cholesterol (HDL-C) level < 40 mg/dL, or a self-reported dyslipidemia. Considering that other meteorological factors may also influence the outcomes, we obtained average temperature and humidity data from 2011 to 2015 for the prefecture-level cities where the participants resided. The annual average temperature data of the participants’ respective prefecture-level cities were obtained from the National Environmental Information Center under the National Oceanic and Atmospheric Administration, whereas the annual average humidity data were sourced from the China Ground Climate Data Daily Dataset (V3.0) provided by the National Meteorological Science Data Center. Inverse distance weighting was applied to interpolate the daily data, generating the corresponding raster data.

### Statistical analysis

Continuous variables are described as the mean and standard deviation (M, SD) or interquartile range (IQR), whereas categorical variables are described as frequency and percentage (n, %). All confounding variables and the corresponding 2011 IR indices were included in the multivariate regression model to create the adjusted model. Subgroup analyses further stratified the study population by sex, age, education level, the place for residence, BMI, and indoor clean fuel usage. Bayesian kernel machine regression (BKMR) and weighted quantile sum (WQS) regression were further used to evaluate the effects of individual and combined exposure to multiple air pollutants on the IR indices (33, 34). BKMR involves the generation of a kernel function based on mixture variables, and the use of Bayesian sampling and analytical methods to produce relationship curves between the mixture components and the outcomes. Additionally, other pollutants can be held at specific quartile concentration levels to examine the effect of a single pollutant on an outcome (35). We grouped the pollutants on basis of the correlation coefficients among the six pollutants and included them in the BKMR model. The number of iterations was set to 10,000 (11). In the WQS model, the dataset was randomly split into training and testing sets. Through maximum likelihood estimation and validation, the weight of each pollutant could be determined, which helped to overcome the multicollinearity issue commonly found in traditional regression methods (33). Mediation analysis was used to explore the mediating effect of BMI. We first assessed the association between air pollution and BMI using multiple linear regression, incorporating relevant covariates to control for confounding factors (age, sex, marital status, insurance coverage, education level, place of residence, cooking fuel usage, nighttime activities, daily physical activity, alcohol consumption status, smoking status, hypertension status, diabetes status, dyslipidaemia status, CRP level, BUN level, air humidity, and average temperature). Mediation analysis was conducted via the R package “mediation,” with the bootstrap method employed to estimate the standard errors of the mediated effects. The number of Monte Carlo simulations was set to 1,000 to obtain more accurate estimates. Finally, we conducted sensitivity analyses: (1) a quantile-based g computation (Qgcomp) model was used to analyze the cumulative effects of mixed pollution pollutant exposure; (11). (2) Patients with malignant tumors were excluded; and (3) patients using antihyperglycaemic medications (oral hypoglycaemic agents or insulin) were excluded (3). For the results of multiple linear regression, the false discovery rate (FDR) method was applied to adjust the *p*-values, reducing the likelihood of false positives. A p-value or FED of less than 0.05 was considered statistically significant. The significance codes were as follows: “***” for a P/FDR value <0.001, “**” for a P/FDR value <0.01, and “*” for a P/FDR value <0.05. All the statistical analyses were conducted using R version 4.4.1.

## Results

### Participant and air pollutant characteristics

**Figure 1:**
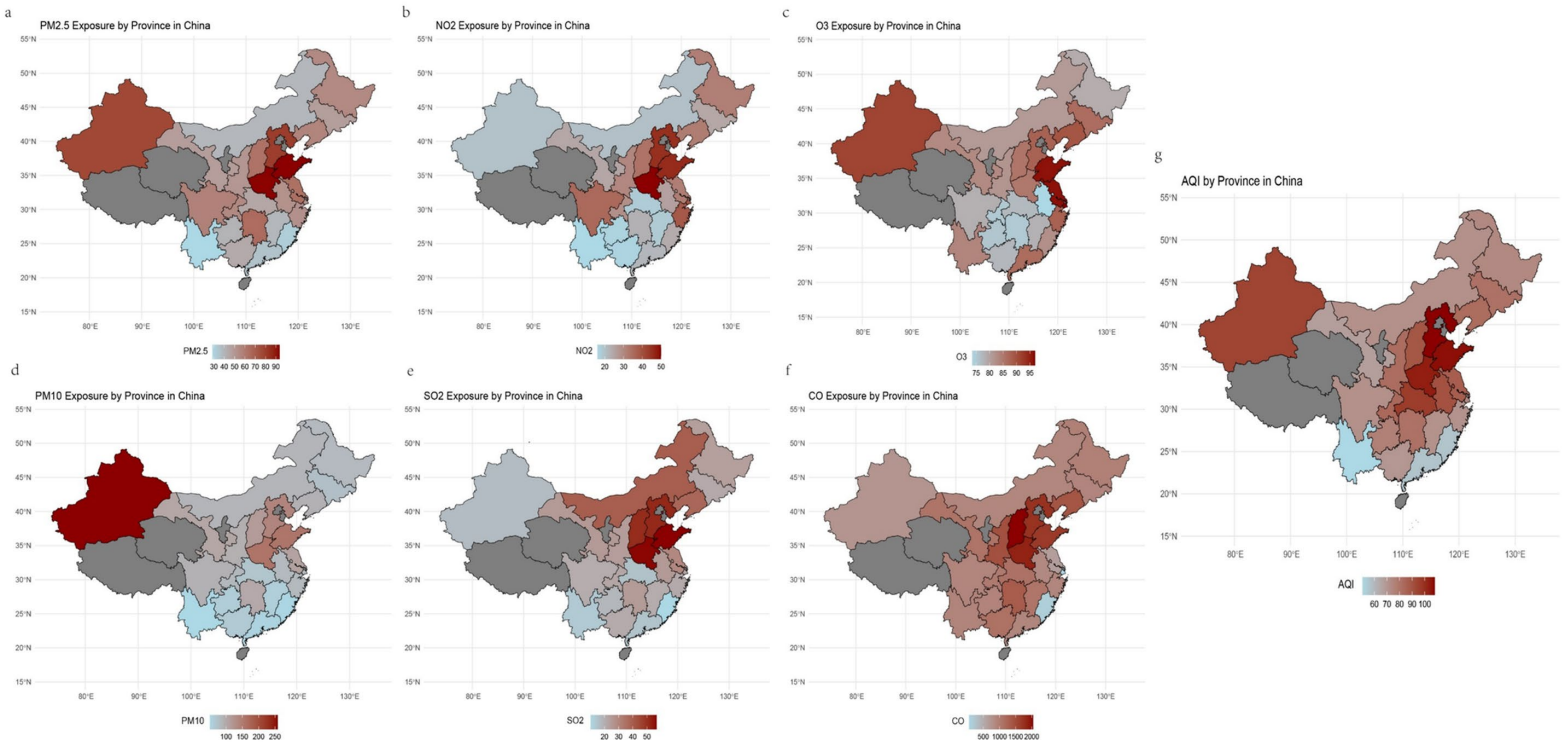
The geographic distribution of the study population and 6 air pollutants, along with AQI, from 2011 to 2015.

A total of 2,219 participants, including 1,060 females and 1,159 males, were ultimately included in our study (Table 1). Statistical differences were observed between the two sexes in terms of age, marital status, place of residence, smoking status, and alcohol consumption status. Figure 1 illustrates the spatial distribution of air pollutants in the provinces where the participants resided. There were significant regional variations in air pollution across China: during the follow-up period, the air quality in Northwest China and North China was notably worse than that in the Southwest and Southeast China. Table 1 also presents the average concentrations (μg/m^3^) of six air pollutants during the follow-up period, revealing no significant differences in air pollution exposure between the two sexes. Additionally, we found that females had higher IR indices than males did (*p* < 0.001).

**Table 1:** Baseline information of participants.

	Levels	Overall	Female	Male	*p*-value
N		2,219	1,060	1,159	
Age [year, mean (SD)]		59.83 (9.07)	58.69 (9.59)	60.87 (8.43)	<0.001
Education (%)	Beyond secondary	2,184 (98.4)	1,047 (98.8)	1,137 (98.1)	0.272
	Secondary or above	35 (1.6)	13 (1.2)	22 (1.9)	
Marry (%)	Married or cohabitation	1713 (77.2)	740 (69.8)	973 (84.0)	<0.001
	Else	506 (22.8)	320 (30.2)	186 (16.0)	
Residence (%)	Rural	1837 (82.8)	834 (78.7)	1,003 (86.5)	<0.001
	Urban	382 (17.2)	226 (21.3)	156 (13.5)	
Medical insurance (%)	No Insurance	116 (5.2)	62 (5.8)	54 (4.7)	0.245
	Under Insurance	2,103 (94.8)	998 (94.2)	1,105 (95.3)	
Annual average expenditure [Yuan, mean (SD)]		11694.77 (39468.41)	11607.77 (39763.33)	11774.33 (39213.74)	0.921
Daily activity level (%)	Low	1,150 (51.8)	446 (42.1)	704 (60.7)	<0.001
	Medium	487 (21.9)	275 (25.9)	212 (18.3)	
	High	582 (26.2)	339 (32.0)	243 (21.0)	
Drinking or not (%)	No	1,467 (66.1)	937 (88.4)	530 (45.7)	<0.001
	Yes	752 (33.9)	123 (11.6)	629 (54.3)	
Smoking or not (%)	No	1,280 (57.7)	988 (93.2)	292 (25.2)	<0.001
	Yes	939 (42.3)	72 (6.8)	867 (74.8)	
Having hypertension or not (%)	No	1,296 (58.4)	601 (56.7)	695 (60.0)	0.129
	Yes	923 (41.6)	459 (43.3)	464 (40.0)	
Having dyslipidemia or not (%)	No	1,224 (55.2)	571 (53.9)	653 (56.3)	0.259
	Yes	995 (44.8)	489 (46.1)	506 (43.7)	
Having diabetes or not (%)	No	2094 (94.4)	987 (93.1)	1,107 (95.5)	0.018
	Yes	125 (5.6)	73 (6.9)	52 (4.5)	
WC [cm, mean (SD)]		85.77 (9.40)	86.60 (9.44)	85.00 (9.30)	<0.001
BMI [kg/m^2^, mean (SD)]		23.61 (3.28)	24.35 (3.39)	22.94 (3.02)	<0.001
Nighttime [hour, mean (SD)]		6.35 (1.88)	6.29 (1.91)	6.39 (1.85)	0.195
CRP [mg/L, mean (SD)]		1.89 (2.74)	1.80 (2.62)	1.97 (2.85)	0.163
BUN [mg/dL, mean (SD)]		15.52 (4.00)	14.79 (3.82)	16.19 (4.06)	<0.001
PM_2.5_ [μg/m^3^, mean (SD)]		57.18 (19.52)	57.61 (19.24)	56.78 (19.77)	0.317
PM_10_ [μg/m^3^, mean (SD)]		96.96 (35.28)	97.92 (35.52)	96.08 (35.06)	0.22
O_3_ [μg/m^3^, mean (SD)]		84.71 (6.89)	84.86 (7.01)	84.56 (6.78)	0.312
NO_2_ [μg/m^3^, mean (SD)]		30.21 (9.97)	30.41 (9.97)	30.02 (9.97)	0.356
SO_2_ [μg/m^3^, mean (SD)]		31.71 (14.49)	32.24 (14.75)	31.22 (14.24)	0.098
CO [μg/m^3^, mean (SD)]		1110.20 (467.07)	1117.33 (468.25)	1103.68 (466.09)	0.492
TyG [mean (SD)]		8.73 (0.62)	8.81 (0.61)	8.65 (0.62)	<0.001
TyG-BMI [mean (SD)]		208.46 (37.02)	216.72 (36.97)	200.91 (35.44)	<0.001
TyG-WC [mean (SD)]		757.93 (116.59)	772.81 (112.92)	744.32 (118.27)	<0.001
METS-IR [mean (SD)]		35.72 (6.72)	36.75 (6.66)	34.78 (6.64)	<0.001
Annual average air humidity [%, mean (SD)]		69.48 (8.08)	69.29 (8.12)	69.65 (8.04)	0.293
Annual average temperature [°C, mean (SD)]		14.90 (4.52)	14.72 (4.60)	15.07 (4.44)	0.068

**Figure 2:**
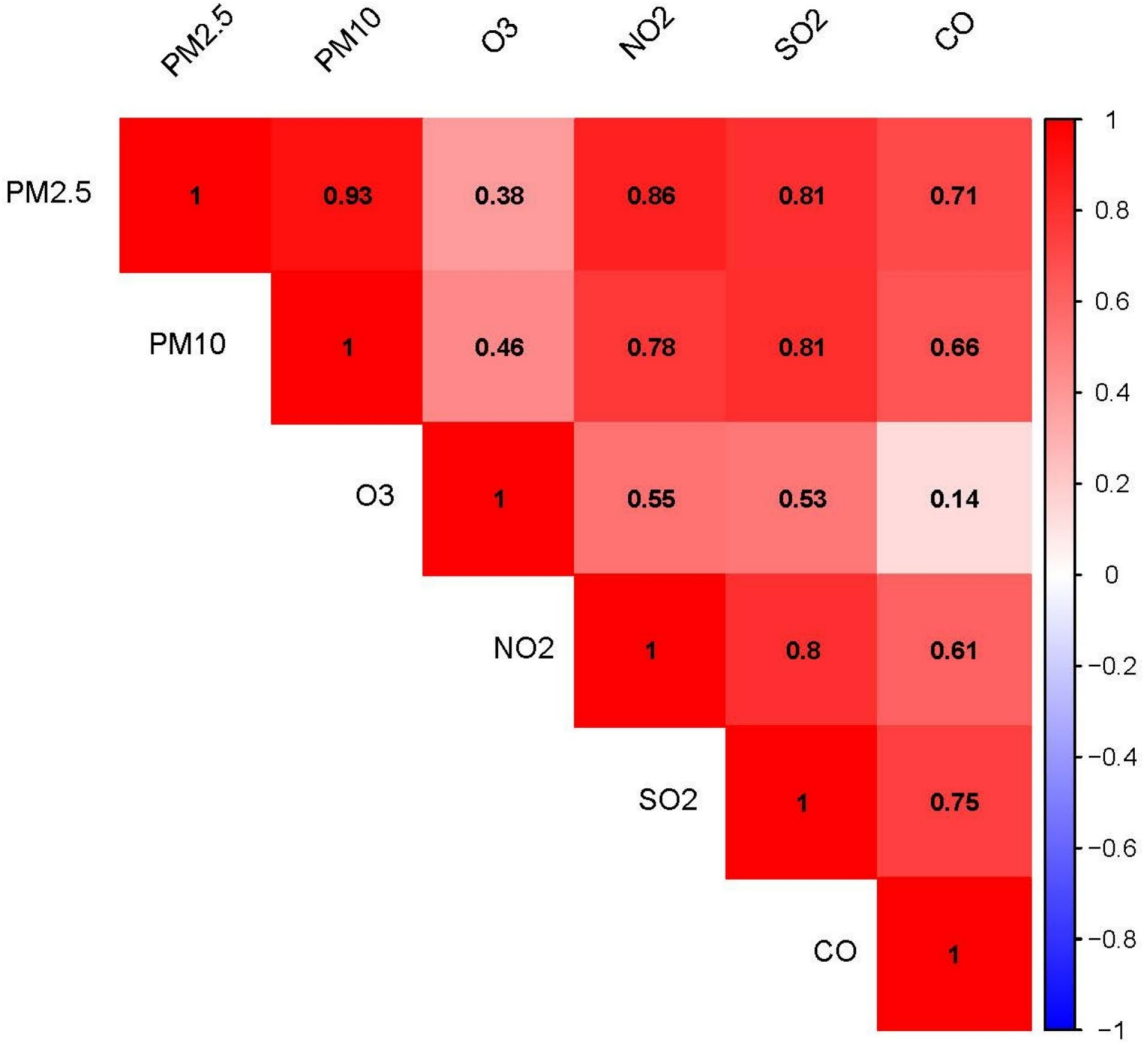
Correlation heatmap of the six air pollutants: The numerical values and color intensity represent the magnitude of the correlation coefficient (r^2^), with red indicating positive correlation and blue indicating negative correlation.

Figure 2 shows the correlation coefficients among the six air pollutants. PM_2.5_ and PM_10_ had the highest correlation coefficient (r^2^ = 0.93). The correlation coefficients between O_3_ and PM_2.5_, PM_10_, and CO were relatively low, while the remaining air pollutants exhibited varying degrees of correlation with each other.

### Linear regression analysis of air pollutants and IR

Table 2 presents the associations between levels of exposure to air pollutants and IR indices as indicated by linear regression analysis. The model indicated that after adjustment for all confounding factors, an increase of 1 μg/m^3^ in the concentrations of PM_2.5_, PM_10_, SO_2_, NO_2_, CO, and O_3_ was associated with a potential increase of 0.155–0.674 in the TyG-WC value, a rise of 0.001–0.029 in the METS-IR value, and an increase of 0.027–0.128 in the TyG-BMI value. Although the results are inconsistent, they still suggest a potential positive association between exposure to air pollutants and IR.

**Table 2:** Results of the multivariable linear regression for different air pollutant exposure concentrations and four IR indices (By FDR).

	TyG [*β*-coefficients(95%CI)]			TyG-WC [β-coefficients(95%CI)]	
	Crude model	Adjusted model		Crude model	Adjusted model
PM_2.5_	0.000 (−0.001, 0.002)	−0.001 (−0.002, 0.001)	PM_2.5_	1.127 (0.882, 1.371)***	0.255 (0.080, 0.431)**
PM_10_	0.000 (−0.000, 0.001)	−0.001 (−0.001, 0.001)	PM_10_	0.661 (0.527, 0.796)***	0.155 (0.043, 0.262)*
SO_2_	0.001 (−0.001, 0.002)	−0.000 (−0.001, 0.001)	SO_2_	1.444 (1.114, 1.774)***	0.309 (0.025, 0.593)*
NO_2_	0.001 (−0.001, 0.004)	−0.000 (−0.003, 0.002)	NO_2_	2.255 (1.776, 2.732)***	0.654 (0.302, 1.005)***
CO	0.000 (−0.000, 0.000)	−0.000 (−0.000, 0.000)	CO	0.032 (0.022, 0.042)***	0.008 (−0.000, 0.016)
O_3_	0.004 (0.001,0.008)*	0.000 (−0.001, 0.001)	O_3_	2.421 (1.723, 3.118)***	0.674 (0.155, 0.193)*

	TyG-BMI [β-coefficients(95%CI)]			METS-IR [*β*-coefficients (95%CI)]	
	Crude model	Adjusted model		Crude model	Adjusted model
PM_2.5_	0.324 (0.246, 0.402)***	0.041 (−0.000, 0.086)	PM_2.5_	0.063 (0.049, 0.077)***	0.011 (0.002, 0.021)*
PM_10_	0.188 (0.144, 0.231)***	0.027 (0.001, 0.052)*	PM_10_	0.037 (0.029, 0.045)***	0.006 (0.001, 0.012)*
SO_2_	0.448 (0.343, 0.553)***	0.083 (0.020, 0.146)*	SO_2_	0.090 (0.071, 0.109)***	0.017 (0.002, 0.031)*
NO_2_	0.660 (0.508, 0.812)***	0.130 (0.041, 0.220)**	NO_2_	0.131 (0.104, 0.159)***	0.029 (0.011, 0.042)**
CO	0.010 (0.006, 0.013)***	0.003 (0.001, 0.005)**	CO	0.002 (0.001, 0.002)***	0.001 (0.001, 0.001)*
O_3_	0.628 (0.405, 0.850)***	0.158 (0.029, 0.286)*	O_3_	0.136 (0.096, 0.176)***	0.022 (0.004, 0.049)

Adjusted Model: Age, sex, marital status, insurance coverage, education level, place of residence, cooking fuel usage, nighttime activities, daily physical activity, alcohol consumption status, smoking status, hypertension status, diabetes status, dyslipidaemia status, CRP level, BUN level, TyG/TyG-BMI/TyG-WC/METS-IR levels in 2011, air humidity, average temperature. ***for a P/FDR value <0.001, **for a P/FDR value <0.01, and *for a P/FDR value <0.05.

### Relationships between concurrent exposure to air pollution and IR

**Figure 3:**
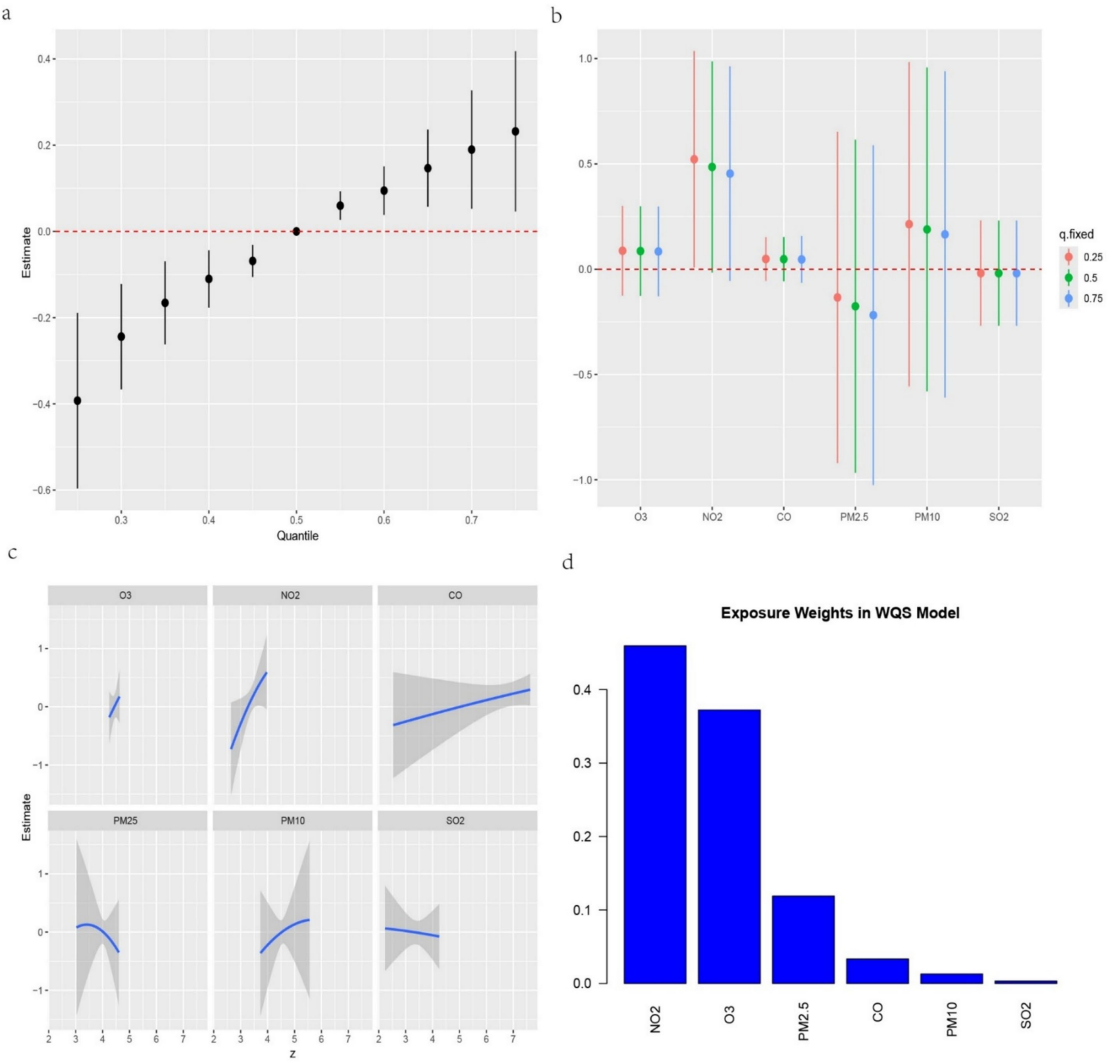
**(a)** Relationship between overall air pollution exposure and METS-IR assessed by BKMR model. **(b)** When other air pollutants were fixed at specific exposure percentiles (25, 50 and 75th), the effect of a particular median air pollutant on METS-IR estimated using BKMR. **(c)** The weight of each of six air pollutants assessed by WQS model. **(d)** The dose–response curves between each air pollutant and METS-IR.

**Figure 4:**
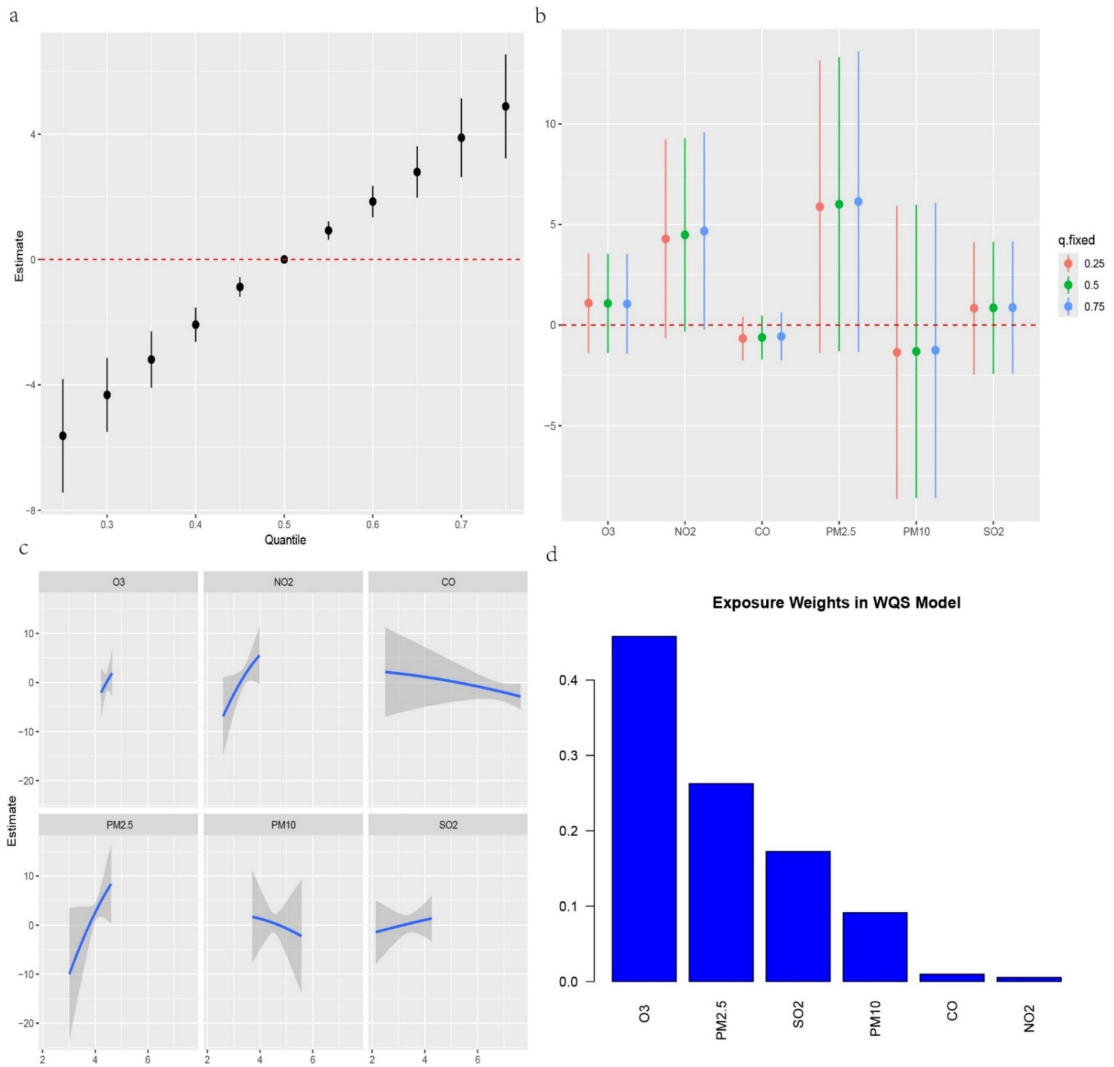
**(a)** Relationship between overall air pollution exposure and TyG-BMI assessed by BKMR model. **(b)** When other air pollutants were fixed at specific exposure percentiles (25, 50 and 75), the effect of a particular median air pollutant on TyG-BMI estimated using BKMR. **(c)**. The weight of each of six air pollutants assessed by WQS model. **(d)** The dose–response curves between each air pollutant and TyG-BMI.

**Figure 5:**
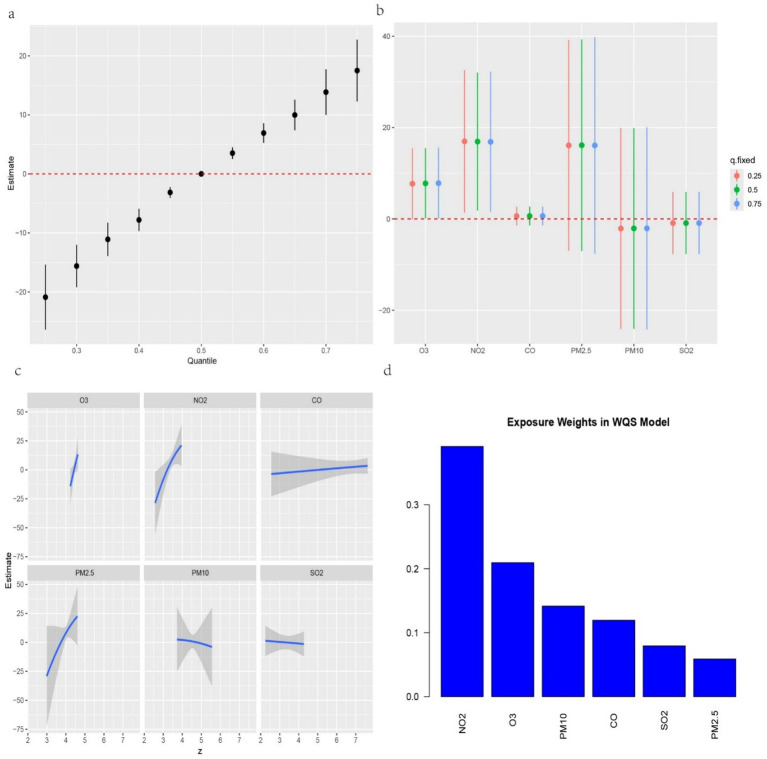
**(a)** Relationship between overall air pollution exposure and TyG-WC assessed by BKMR model. **(b)**. When other air pollutants were fixed at specific exposure percentiles (25, 50 and 75th), the effect of a particular median air pollutant on TyG-WC estimated using BKMR. **(c)**. The weight of each of six air pollutants assessed by WQS model. **(d)** The dose–response curves between each air pollutant and TyG-WC.

We further investigated the associations between the three IR indices—METS-IR, TyG-BMI and TyG-WC—and the six air pollutants using mixture models (Figures 3–5). BKMR also revealed a significant positive correlation between the NO₂ concentration and the an increase in the METS-IR and TyG-WC when the concentrations of other air pollutants were held at the 25th percentile. At the 50th percentile, the NO₂ concentration was positively correlated with the an increase in the TyG-BMI and TyG-WC, whereas at the 75th percentile, the NO₂ concentration was only positively correlated with only the an increase in the TyG-WC. The O₃ concentration was positively correlated with an increase in the TyG-WC when the concentrations of other air pollutants were held between the 25th and 75th percentiles. The WQS model, in which the weights of the six pollutants in the mixture were calculated, revealed that NO₂ and O₃ consistently had relatively high weights, indicating their predominant influence on IR. Dose–response curve analysis indicated that the effects of both NO₂ and O₃ exposure on IR were linear and positively correlated (Figures 3–5). Association between air pollution exposure and IR indices analyzed using multiple models.

### Analysis of the mediating effect of BMI

Multiple linear regression revealed a consistent positive correlation between air pollution exposure and BMI (Supplementary Table 2). We conducted a mediation analysis based on adjusted linear models to investigate whether BMI potentially mediated the association between air pollution exposure and IR. As shown in Table 3, BMI significantly mediated the association between exposure to the six air pollutants and IR, with mediation proportions ranging from 49.1%–93.5% (FDR < 0.05). Additionally, we found that the effect of air pollution indicators on IR was primarily mediated by BMI, rather than through a direct effect (FDR for ACME <0.05 while FDR for ADE > 0.05).

**Table 3:** Mediating role of BMI between air pollutant exposure concentrations and IR scores (By adjusted model).

M = BMI		ACME		ADE		Prop. Mediated(95%CI)	FDR
X	Y	Estimate(95%CI)	FDR	Estimate(95%CI)	FDR		
PM_2.5_	METS-IR	0.022 (0.016, 0.030)	**<2e-16**	0.006 (−0.003, 0.010)	0.33	0.792 (0.590, 1.170)	**<2e-16**
PM_10_		0.013 (0.009, 0.020)	**<2e-16**	0.003 (−0.002, 0.010)	0.37	0.799 (0.572, 1.220)	**<2e-16**
SO_2_		0.032 (0.023, 0.040)	**<2e-16**	0.008 (−0.005, 0.020)	0.36	0.794 (0.568, 1.190)	**<2e-16**
NO_2_		0.047 (0.034, 0.060)	**<2e-16**	0.017 (0.001, 0.030)	0.12	0.732 (0.546, 0.980)	**<2e-16**
CO		0.000 (0.000, 0.001)	**<2e-16**	0.000 (0.000, 0.001)	0.12	0.533 (0.311, 0.950)	**<2e-16**
R O_3_		0.033 (0.019, 0.050)	**<2e-16**	0.018 (−0.006, 0.040)	0.27	0.651 (0.378, 1.220)	**<2e-16**
PM_2.5_	TyG-BMI	0.124 (0.087, 0.160)	**<2e-16**	0.009 (−0.034, 0.060)	0.81	0.935 (0.671, 1.510)	**<2e-16**
PM_10_		0.071 (0.049, 0.090)	**<2e-16**	0.001 (−0.029, 0.030)	0.97	0.991 (0.692, 1.730)	**<2e-16**
SO_2_		0.179 (0.127, 0.230)	**<2e-16**	0.009 (−0.067, 0.090)	0.86	0.952 (0.650, 1.580)	**<2e-16**
NO_2_		0.263 (0.189, 0.350)	**<2e-16**	0.052 (−0.038, 0.150)	0.39	0.836 (0.629, 1.170)	**<2e-16**
CO		0.001 (0.000. 0.002)	**<2e-16**	0.005 (0.002, 0.010)	0.12	0.579 (0.354, 1.030)	**<2e-16**
O_3_		0.185 (0.104, 0.270)	**<2e-16**	0.233 (0.088, 0.380)	0.11	0.796 (0.438, 1.720)	0.06
PM_2.5_	TyG-WC	0.308 (0.226, 0.400)	**<2e-16**	0.159 (−0.000, 0.320)	0.12	0.659 (0.457, 1.000)	**<2e-16**
PM_10_		0.176 (0.126, 0.230)	**<2e-16**	0.103 (0.006, 0.210)	0.12	0.632 (0.439, 0.970)	**<2e-16**
SO_2_		0.447 (0.321, 0.570)	**<2e-16**	0.137 (−0.140, 0.380)	0.43	0.765 (0.519, 1.480)	**<2e-16**
NO_2_		0.653 (0.493, 0.820)	**<2e-16**	0.413 (0.081, 0.740)	0.12	0.613 (0.448, 0.900)	**<2e-16**
CO		0.007 (0.004, 0.010)	**<2e-16**	0.007 (−0.001, 0.010)	0.16	0.491 (0.269, 1.140)	**<2e-16**
O_3_		0.465 (0.270, 0.680)	**<2e-16**	0.532 (0.036, 1.030)	0.12	0.467 (0.266, 0.930)	**<2e-16**

ACME, Average Causal Mediation Effect. ADE, Average Direct Effect. Prop. Mediated, Proportion of Mediation. M, Mediation variable. Bold values indicate statistically significant results (P/FDR < 0.05).

### Stratification of air pollution exposure in relation to IR

The relationships between exposure to air pollutants and IR indices stratified by age, gender, education level, residence, BMI, and cooking fuel type were shown in Tables 4–6. After adjusting for potential confounders, we detected a significant effect of the interaction effect between individuals with a BMI less than 24 kg/m^2^ and those with a BMI greater than 24 kg/m^2^ in the subgroup analysis of O3’sthe effect of O3 exposure on the TyG-BMI. Overall, stronger associations between air pollution exposure and IR were observed in the subgroups of males, individuals with an education levels below a secondary level, those individuals living in rural areas, individuals those using clean energy for cooking, individuals with a BMI ≤ 24 kg/m^2^, and those individuals aged ≤65 years.

**Table 4:** Subgroup analyses of air pollutant exposure concentrations and METS-IR (By adjusted model).

	N (%)	PM_2.5_			PM_10_			O_3_		
		β (95%CI)	FDR	P for interaction	β (95%CI)	FDR	P for interaction	β (95%CI)	FDR	P for interaction
Gender				0.47			0.37			0.92
Female	1,060 (47.77)	0.01 (−0.01–0.02)	0.50		0.00 (−0.01–0.01)	0.73		0.02 (−0.02–0.05)	0.52	
Male	1,159 (52.23)	0.02 (0.01–0.03)	0.06		0.01 (0.01–0.02)	**0.04**		0.03 (−0.01–0.06)	0.41	
Education				0.41			0.36			0.89
Secondary-	2,184 (98.42)	0.01 (0.01–0.02)	0.06		0.01 (0.01–0.01)	0.07		0.02 (−0.00–0.05)	0.32	
Secondary+	35 (1.58)	0.04 (−0.06–0.14)	0.42		0.02 (−0.04–0.09)	0.61		0.05 (−0.23–0.34)	0.86	
Residence				0.30			0.07			0.20
Rural	1837 (82.79)	0.01 (0.01–0.02)	0.50		0.01 (0.01–0.01)	0.05		0.03 (−0.00–0.06)	0.24	
Urban	382 (17.21)	0.00 (−0.02–0.03)	0.06		−0.00 (−0.02–0.01)	0.61		0.01 (−0.05–0.07)	0.86	
Cooking fuel type				0.74			0.56			0.46
Clean fuel	1,220 (54.98)	0.01 (−0.00–0.02)	0.82		0.01 (−0.00–0.01)	0.30		0.03 (−0.00–0.07)	0.24	
Non-clean fuel	999 (45.02)	0.01 (−0.00–0.02)	0.17		0.01 (−0.00–0.01)	0.23		0.00 (−0.04–0.04)	0.93	
BMI				0.37			0.45			0.09
≤24	1,224 (55.16)	0.01 (−0.00–0.02)	0.26		0.01 (−0.00–0.01)	0.16		0.05 (0.02–0.08)	**0.04**	
> 24	995 (44.84)	−0.01 (−0.02–0.01)	0.50		−0.01 (−0.01–0.00)	0.32		−0.02 (−0.05–0.02)	0.52	
Age				0.35			0.35			0.83
≤65	1,131 (50.97)	0.01 (0.01–0.03)	**0.03**		0.01 (0.01–0.02)	0.11		0.02 (−0.02–0.06)	0.43	
> 65	1,088 (49.03)	0.01 (−0.00–0.02)	0.29		0.01 (−0.00–0.01)	0.32		0.02 (−0.02–0.06)	0.43	

	N (%)	NO_2_			SO_2_			CO		
		β (95%CI)	FDR	P for interaction	β (95%CI)	FDR	P for interaction	β (95%CI)	FDR	P for interaction
Gender				0.70			0.61			0.93
Female	1,060 (47.77)	0.02 (−0.01–0.05)	0.20		0.01 (−0.02–0.03)	0.63		0.00 (−0.00–0.00)	0.26	
Male	1,159 (52.23)	0.04 (0.01–0.06)	0.02		0.03 (0.01–0.05)	0.09		0.00 (0.01–0.01)	0.07	
Education				0.62			0.88			0.23
Secondary-	2,184 (98.42)	0.03 (0.01–0.05)	0.01		0.02 (0.01–0.03)	0.09		0.00 (0.01–0.01)	0.07	
Secondary+	35 (1.58)	0.05 (−0.09–0.19)	0.62		0.03 (−0.09–0.15)	0.63		0.00 (−0.00–0.01)	0.17	
Residence				0.30			0.46			0.77
Rural	1837 (82.79)	0.03 (0.01–0.05)	0.01		0.02 (0.01–0.03)	0.09		0.00 (0.01–0.01)	0.07	
Urban	382 (17.21)	0.01 (−0.04–0.06)	0.68		0.01 (−0.02–0.05)	0.57		0.00 (−0.00–0.00)	0.23	
Cooking fuel type				0.91			0.73			0.90
Clean fuel	1,220 (54.98)	0.03 (0.01–0.06)	0.02		0.02 (0.01–0.04)	0.09		0.00 (0.01–0.01)	0.07	
Non-clean fuel	999 (45.02)	0.02 (−0.01–0.05)	0.18		0.01 (−0.01–0.03)	0.51		0.00 (−0.00–0.00)	0.17	
BMI				0.59			0.98			0.13
≤24	1,224 (55.16)	0.02 (−0.00–0.04)	0.18		0.01 (−0.01–0.03)	0.44		0.00 (−0.00–0.00)	0.71	
> 24	995 (44.84)	−0.00 (−0.03–0.03)	0.94		−0.01 (−0.03–0.01)	0.45		0.00 (−0.00–0.00)	0.23	
Age				0.57			0.87			0.14
≤65	1,131 (50.97)	0.02 (−0.00–0.04)	0.18		0.01 (−0.01–0.03)	0.33		0.00 (0.01–0.01)	0.07	
> 65	1,088 (49.03)	0.04 (0.01–0.07)	**0.01**		0.02 (−0.00–0.04)	0.13		0.00 (−0.00–0.00)	0.26	

Bold values indicate statistically significant results (P/FDR < 0.05).

**Table 5:** Subgroup analyses of air pollutant exposure concentrations and TyG-BMI (By adjusted model).

		PM_2.5_			PM_10_			O_3_		
	N (%)	β (95%CI)	FDR	P for interaction	β (95%CI)	FDR	P for interaction	β (95%CI)	FDR	P for interaction
Gender				0.30			0.34			0.95
Female	1,060 (47.77)	−0.01 (−0.09–0.06)	0.83		0.02 (−0.02–0.06)	0.55		0.17 (−0.02–0.36)	0.15	
Male	1,159 (52.23)	0.05 (−0.02–0.11)	0.63		0.03 (−0.00–0.07)	0.19		0.13 (−0.04–0.31)	0.21	
Education				0.63			0.71			0.94
Secondary-	2,184 (98.42)	0.02 (−0.03–0.07)	0.63		0.03 (0.01–0.05)	0.17		0.16 (0.03–0.29)	**0.03**	
Secondary+	35 (1.58)	0.19 (−0.31–0.69)	0.63		0.20 (−0.09–0.50)	0.39		0.41 (−0.99–1.82)	0.57	
Residence				0.40			0.27			0.30
Rural	1837 (82.79)	0.03 (−0.03–0.08)	0.63		0.03 (0.01–0.06)	0.17		0.18 (0.03–0.32)	**0.03**	
Urban	382 (17.21)	−0.05 (−0.18–0.08)	0.63		0.00 (−0.05–0.06)	0.97		0.11 (−0.17–0.39)	0.49	
Cooking fuel type				0.38			0.31			0.72
Clean fuel	1,220 (54.98)	0.01 (−0.06–0.08)	0.87		0.02 (−0.02–0.06)	0.46		0.16 (−0.02–0.34)	0.15	
Non-clean fuel	999 (45.02)	0.04 (−0.04–0.11)	0.63		0.03 (−0.00–0.07)	0.19		0.16 (−0.02–0.35)	0.15	
BMI				0.14			0.41			**0.01**
≤24	1,224 (55.16)	0.02 (−0.03–0.07)	0.63		0.02 (−0.01–0.05)	0.39		0.23 (0.09–0.38)	**0.01**	
> 24	995 (44.84)	−0.07 (−0.14–0.01)	0.63		−0.01 (−0.04–0.03)	0.76		−0.07 (−0.25–0.11)	0.49	
Age				0.24			0.27			0.45
≤65	1,131 (50.97)	0.04 (−0.02–0.11)	0.63		0.05 (0.01–0.08)	0.17		0.23 (0.04–0.41)	**0.03**	
> 65	1,088 (49.03)	−0.00 (−0.07–0.07)	0.98		0.01 (−0.03–0.04)	0.82		0.08 (−0.10–0.26)	0.49	

	N (%)	NO_2_			SO_2_			CO		
		β (95%CI)	FDR	P for interaction	β (95%CI)	FDR	P for interaction	β (95%CI)	FDR	P for interaction
Gender				0.65			0.44			0.75
Female	1,060 (47.77)	0.11 (−0.03–0.24)	0.17		0.06 (−0.03–0.16)	0.32		0.00 (−0.00–0.01)	0.10	
Male	1,159 (52.23)	0.14 (0.02–0.26)	0.08		0.09 (0.01–0.18)	0.09		0.00 (0.01–0.01)	0.05	
Education				0.69			0.73			0.13
Secondary-	2,184 (98.42)	0.13 (0.04–0.22)	**0.03**		0.08 (0.02–0.15)	0.06		0.00 (0.01–0.01)	**0.02**	
Secondary+	35 (1.58)	0.25 (−0.50–0.99)	0.63		0.26 (−0.29–0.82)	0.44		0.02 (0.01–0.03)	0.06	
Residence				0.27			0.44			0.29
Rural	1837 (82.79)	0.14 (0.04–0.24)	**0.03**		0.09 (0.02–0.16)	0.06		0.00 (0.01–0.01)	0.05	
Urban	382 (17.21)	0.03 (−0.19–0.25)	0.88		0.05 (−0.10–0.20)	0.57		0.01 (0.01–0.01)	0.06	
Cooking fuel type				0.47			0.81			0.94
Clean fuel	1,220 (54.98)	0.12 (−0.00–0.25)	0.10		0.09 (−0.00–0.18)	0.12		0.00 (0.01–0.01)	0.05	
Non-clean fuel	999 (45.02)	0.14 (0.01–0.27)	0.10		0.08 (−0.01–0.17)	0.18		0.00 (0.01–0.01)	0.07	
BMI				0.39			0.68			0.30
≤24	1,224 (55.16)	0.07 (−0.03–0.17)	0.21		0.04 (−0.04–0.11)	0.41		0.00 (−0.00–0.00)	0.61	
> 24	995 (44.84)	0.01 (−0.12–0.13)	0.92		−0.01 (−0.09–0.08)	0.86		0.00 (−0.00–0.01)	0.07	
Age				0.90			0.68			0.25
≤65	1,131 (50.97)	0.13 (0.01–0.26)	0.10		0.11 (0.02–0.20)	0.06		0.00 (0.01–0.01)	**0.02**	
> 65	1,088 (49.03)	0.13 (−0.00–0.26)	0.10		0.05 (−0.04–0.14)	0.37		0.00 (−0.00–0.00)	0.22	

Bold values indicate statistically significant results (P/FDR < 0.05).

**Table 6:** Subgroup analyses of air pollutant exposure concentrations and TyG-WC (By adjusted model).

	N (%)	PM_2.5_			PM_10_			O_3_		
		β (95%CI)	FDR	P for interaction	β (95%CI)	FDR	P for interaction	β (95%CI)	FDR	P for interaction
Gender				0.66			0.63			0.92
Female	1,060 (47.77)	0.22 (−0.04–0.49)	0.15		0.12 (−0.04–0.28)	0.20		0.72 (−0.04–1.48)	0.13	
Male	1,159 (52.23)	0.27 (0.03–0.51)	0.06		0.17 (0.02–0.32)	0.05		0.61 (−0.11–1.33)	0.15	
Education				0.53			0.88			0.43
Secondary-	2,184 (98.42)	0.26 (0.09–0.44)	**0.02**		0.15 (0.04–0.26)	**0.02**		0.65 (0.13–1.18)	0.07	
Secondary+	35 (1.58)	0.91 (−1.26–3.08)	0.51		1.34 (−0.17–2.86)	0.17		2.29 (−4.09–8.68)	0.54	
Residence				0.34			0.20			0.99
Rural	1837 (82.79)	0.29 (0.10–0.48)	**0.02**		0.19 (0.07–0.31)	**0.02**		0.70 (0.12–1.27)	0.07	
Urban	382 (17.21)	−0.09 (−0.59–0.40)	0.78		−0.12 (−0.41–0.16)	0.44		0.62 (−0.61–1.86)	0.39	
Cooking fuel type				0.58			0.55			0.74
Clean fuel	1,220 (54.98)	0.23 (−0.02–0.47)	0.11		0.12 (−0.03–0.27)	0.18		0.58 (−0.11–1.26)	0.15	
Non-clean fuel	999 (45.02)	0.28 (0.02–0.55)	0.07		0.18 (0.02–0.35)	0.05		0.88 (0.06–1.70)	0.09	
BMI				0.33			0.71			0.33
≤24	1,224 (55.16)	0.28 (0.06–0.49)	**0.03**		0.19 (0.06–0.33)	**0.02**		1.09 (0.44–1.74)	**0.01**	
> 24	995 (44.84)	−0.02 (−0.30–0.25)	0.86		−0.04 (−0.21–0.13)	0.68		0.07 (−0.67–0.82)	0.85	
Age				0.25			0.26			0.59
≤65	1,131 (50.97)	0.33 (0.10–0.56)	**0.02**		0.20 (0.05–0.34)	**0.02**		0.77 (0.05–1.49)	**0.04**	
> 65	1,088 (49.03)	0.20 (−0.07–0.47)	0.20		0.11 (−0.05–0.28)	0.22		0.57 (−0.18–1.33)	0.18	

	N (%)	NO_2_			SO_2_			CO		
		β (95%CI)	FDR	P for interaction	β (95%CI)	FDR	P for interaction	β (95%CI)	FDR	P for interaction
Gender				0.83			0.84			0.76
Female	1,060 (47.77)	0.64 (0.11–1.16)	**0.02**		0.26 (−0.16–0.67)	0.34		0.01 (−0.01–0.02)	0.44	
Male	1,159 (52.23)	0.64 (0.16–1.12)	**0.02**		0.35 (−0.05–0.74)	0.19		0.01 (−0.00–0.02)	0.24	
Education				0.82			0.69			0.22
Secondary-	2,184 (98.42)	0.66 (0.31–1.02)	**0.00**		0.31 (0.02–0.60)	0.14		0.01 (−0.00–0.02)	0.24	
Secondary+	35 (1.58)	0.53 (−2.63–3.69)	0.75		0.27 (−2.38–2.92)	0.84		0.04 (−0.03–0.11)	0.41	
Residence				0.69			0.86			0.60
Rural	1837 (82.79)	0.68 (0.30–1.06)	**0.00**		0.35 (0.04–0.66)	0.14		0.01 (−0.00–0.02)	0.24	
Urban	382 (17.21)	0.25 (−0.72–1.23)	0.66		0.09 (−0.64–0.82)	0.84		0.01 (−0.02–0.03)	0.59	
Cooking fuel type				0.76			0.82			0.83
Clean fuel	1,220 (54.98)	0.65 (0.16–1.14)	**0.02**		0.25 (−0.16–0.66)	0.34		0.01 (−0.00–0.02)	0.31	
Non-clean fuel	999 (45.02)	0.65 (0.13–1.17)	**0.02**		0.35 (−0.06–0.75)	0.19		0.01 (−0.01–0.02)	0.41	
BMI				0.56			0.96			0.89
≤24	1,224 (55.16)	0.58 (0.16–1.01)	**0.02**		0.34 (−0.01–0.70)	0.18		0.01 (−0.00–0.02)	0.25	
> 24	995 (44.84)	0.15 (−0.39–0.70)	0.66		−0.22 (−0.64–0.20)	0.41		0.00 (−0.01–0.01)	0.87	
Age				0.97			0.31			0.22
≤65	1,131 (50.97)	0.59 (0.13–1.06)	**0.02**		0.43 (0.05–0.81)	**0.01**		0.01 (−0.00–0.02)	0.24	
> 65	1,088 (49.03)	0.78 (0.25–1.32)	**0.02**		0.21 (−0.22–0.64)	0.41		0.01 (−0.01–0.02)	0.41	

Bold values indicate statistically significant results (P/FDR < 0.05).

### Sensitivity analysis

First, we examined the associations between air pollution mixtures and IR indices via the Qgcomp model, and similar conclusions were drawn from the BKMR and WQS models. Supplementary Table 3 presents the estimates, standard errors, and *p*-values for each IR indicator. Second, as shown in Supplementary Tables 4, 5, after excluding participants with self-reported malignancies (*N* = 2,196) and those on antidiabetic medications (*N* = 2099), the results of the linear regression analysis remained consistent with the primary findings, thereby enhancing the robustness of the study.

## Discussion

This study investigated the relationships between mixed exposure to six air pollutants—PM_2.5_, PM_10_, SO_2_, NO_2_, CO, and O_3_—and IR indices in a cohort of middle-aged and older Chinese individuals over a four-year period. Owing to age-related metabolic changes, increased fat accumulation, and comorbidities such as hypertension, obesity, and dyslipidemia, which all increase the risk of IR, we selected individuals aged 45 years and above for our study. By focusing on this high-risk group, we aimed to better understand the specific risks and mechanisms of air pollution exposure in individuals who are more susceptible to these conditions. This cohort choice allowed for a deeper investigation into air pollution’s role in metabolic health, particularly among high-risk individuals. Our findings indicated that both individual and combined exposure to these pollutants were significantly associated with increased IR, suggesting a complex and cumulative effect of air pollution on metabolic health. The mixed effect was driven primarily by NO_2_ and O_3_. Additionally, our study demonstrated the mediating effect of BMI on this causal relationship. The sensitivity analysis further confirmed our findings. Our study is one of the few epidemiological studies based on a Chinese cohort to explore the relationship between exposure to air pollutants and IR. These findings support the hypothesis that air pollution exposure has an adverse effect on IR.

Previous studies have confirmed the associations between exposure to air pollution and adverse health outcomes. A study by Jalali et.al (34) that included an Iranian cohort revealed that for every 10 μg/m^3^ increase in PM_2.5_ exposure, the incidence of cardiovascular disease increased by 3% (95% CI = 1.016, 1.036). A cross-sectional study by VoPham et.al (36) also revealed that higher environmental PM_2.5_ exposure was associated with an increased likelihood of nonalcoholic fatty liver disease among hospitalized patients in the United States. Similarly, extensive studies have investigated the association between PM exposure and IR (36–39). Li et al. (37) conducted *in vivo* and *in vitro* experiments, which revealed that mice exposed to O3 exhibited presented increased glucose loads and impaired telomere homeostasis. Our study highlights the differential effects of various pollutants on IR. Furthermore, we initially found that PM_10_ exposure had a stronger positive effect on IR than did PM_2.5_ exposure in both the linear regression and the mixture models. However, upon reanalysis with the BKMR model, we observed that PM_2.5_ exposure had a more significant contribution to the cumulative exposure effect on IR. We hypothesize that the diameter of fine PM may be one of the main reasons for this difference (40). Recent evidence suggests that the diameter of fine PM is a key factor in determining whether extrapulmonary translocation occurs. Extrapulmonary translocation involves that fine PM, moving from the lungs into the bloodstream through the mediation of macrophages, potentially causing greater damage to cardiovascular function (41). However, the results of observational studies are often inconsistent. A meta-analysis shows that compared to PM_2.5_, PM_10_ exposure had a higher relative risk for diabetes (1.26 vs. 1.16) (42). We speculate that the larger particles of PM_10_ may remain in the respiratory tract for a longer duration and could induce a stronger immune response, oxidative stress, and other effects. Further experimental as well as epidemiological studies are needed to elucidate the mechanisms involved.

The biological mechanisms underlying the impact of air pollutants on IR remain difficult to elucidate. Several hypotheses have been proposed by researchers: some studies have suggested that exposure to air pollution might affect insulin function by inducing inflammatory responses and oxidative stress (11–14), and interference with energy metabolism could be another possible mechanism. In mouse model, Rajagopalan et al. (43) revealed that exposure to high concentrations of PM_2.5_ impaired energy expenditure in brown adipose tissue and 18FDG-PET uptake. Their transcriptomic analysis also indicated that air pollution has an epigenetic impact on biological systems. Recent evidence suggested that air pollution exposure could also influence the development of metabolic syndrome through the mediation of the gut microbiota (38).

Notably, while all six pollutants showed associations with the IR indices, the strength of these associations varied. This underscores the need for integrated air quality management strategies that consider the combined effects of multiple pollutants, rather than focusing on individual pollutants in isolation. In real-world conditions, individuals experience simultaneous exposure to multiple air pollutants rather than a single pollutant (15). Epidemiological evidence on the combined effects of exposure to air pollution mixtures on IR in the general population remains limited. In this study, to assess the mixed effects of all six air pollutants on IR more accurately, we applied three different statistical approaches: BKMR, WQS regression and Qgcomp. These methods captured complex pollutant interactions, offering a clearer understanding of their combined health effects. A common feature of these three methods is their ability to handle nonlinear and interaction effects in multivariate exposure data through different functions, making them particularly suitable for analyzing complex environmental exposures (44, 45). Dong et al. (15) used the BKMR and WQS regression to investigate the associations between mixed exposure to PM_1_, PM_2.5_, PM_10_, NO_2_, and O_3_ and the risk of sarcopenia in middle-aged and older individuals. He et al. (46) applied the same methods and revealed consistent positive correlation between combined exposure to heavy metals and the incidence of stroke cases, with lead exposure being the dominant factor driving the mixed effect. BKMR helped to reveal the complexity of the nonlinear relationships and interactions among pollutants. Additionally, WQS regression quantified the contribution of each pollutant to IR, thereby highlighting the dominant role of NO_2_ in mixed exposures, providing strong support for understanding the relative importance of pollutants. Finally, Qgcomp, as a complement to the above two approaches, further validated the heterogeneous effects observed. Compared with single-pollutant analyses, the mixed-exposure analyses provide a more comprehensive understanding compared to single pollutant analyses, highlighting the joint effects of multiple pollutants. These findings carry important public health implications, suggesting the need to pay greater attention to the combined effects of pollutant exposure on health. They also emphasize the necessity of considering the synergistic effects of pollutants when developing air quality management policies.

Our mediation analysis further confirms BMI’s mediating role between air pollution and IR. Given BMI’s established link to metabolic disorders, our findings suggest that air pollution influences IR through its effects on body composition. Air pollutants have been confirmed to be associated with the onset and progression of obesity (47, 48). The use of a broader range of statistical methods has also provided causal evidence for the impact of air pollution on obesity, which is consistent with the findings of other researchers. Studies have reported that air pollution can trigger a systemic chronic inflammatory response, which is closely related to obesity. We hypothesize that air pollutants may activate the immune system, where these cytokines not only induce inflammation but also promote fat accumulation, leading to obesity (49). Additionally, chronic inflammation may alter the secretion function of adipocytes, contributing to weight gain and the onset of IR. In addition, our subgroup analysis results indicate that the impact of air pollution exposure on IR is more significant in males, individuals with an education level below secondary level, individuals living in rural areas and using clean energy for cooking, and with a BMI ≤ 24 and aged ≤65 years. We propose the following hypothesis to better explain these findings: first, males or younger middle-aged individuals may systematically engage in more outdoor work, leading to higher levels of exposure (46). The protective effect of endogenous estrogen may also contribute to the observed sex differences (50). Individuals with lower education levels and those living in rural areas may have poorer health management, imbalanced nutritional intake, or a lack of awareness regarding the hazards of air pollution, increasing their vulnerability to the effects of air pollution exposure. In populations with a BMI ≤ 24, there is no obvious indication of obesity. Individuals with lower BMI may have lower muscle mass levels, and muscle mass plays a crucial role in maintaining blood glucose control. Air pollution may affect insulin sensitivity through related pathways, and this impact could be more pronounced in individuals with lower muscle mass, who have greater difficulty maintaining glucose homeostasis (50, 53). As for individuals using clean energy for cooking, we hypothesize that, on the one hand, they may take more proactive health management measures regarding indoor air quality, leading them to underestimate the risks of outdoor environmental pollution (51). On the other hand, their overall environmental exposure may not have significantly improved, and they might even be more affected by external air pollution because they live in more polluted areas. Therefore, this group may be more sensitive to external pollution. The findings from some subgroup analyses in our study may be inconsistent with those of previous studies. Further social, psychological, and medical interventions need to be implemented to increase the adaptability of vulnerable populations to air pollution.

Our study, which was based on a cohort of middle-aged and older participants in China, utilized data from a four-year follow-up period. This large-scale, long-term longitudinal design effectively captured the long-term effects of air pollution on IR, overcoming the time-effect limitations inherent in cross-sectional studies. We analyzed the impact of six air pollutants on IR, providing a more accurate reflection of real-world exposure to pollutants. The mixed-effect analysis helps to reveal the interactions between different pollutants, enhancing the external validity and reliability of our findings. This discovery has significant public health implications, offering new evidence to policymakers and highlighting the importance of controlling air pollution, particularly by reducing the concentrations of major pollutants, such as NO_2_, to prevent metabolic diseases in middle-aged and older individuals. Moreover, the identification of BMI as a mediating factor provides new directions for health management and intervention.

However, the following limitations should be equally considered: Some limitations arise from the availability of data in the CHARLS database. Owing to the design aimed at protecting participants’ privacy, we could only estimate the annual average air pollution exposure at the administrative level of the participants’ locations, which might introduce some bias in individual exposure estimation. Some studies have suggested that individuals living farther from city centers are more likely to be exposed to higher levels of air pollution, as they spend more time commuting. Relying solely on the average pollutant concentrations in the prefecture-level city of the participants’ residence as the overall exposure measure may overlook this issue. We calculated the participants’ daily activity scores on the basis of the household questionnaire used in the CHARLS to assess their activity levels. This score primarily serves as a retrospective subjective measure and cannot determine whether the participants mainly engage in indoor or outdoor activities. This limitation should be considered given that outdoor physical activities can increase pollutant inhalation and exacerbate harm, depending on the pollutant concentration and exercise intensity (52). A key limitation of our study is the lack of covariates related to the daily dietary patterns in the CHARLS, as well as the absence of other indicators of IR, such as blood insulin levels. We calculated different IR indices on the basis of the available data. Although these indices can represent an individual’s IR level to some extent, the hyperinsulinaemic–euglycaemic clamp is still regarded as the gold standard for assessing IR (18, 19). Due to the differing focuses of the various IR indices—for example, the TyG-BMI and TyG-WC place more emphasis on body measurements, whereas the METS-IR takes fasting blood glucose levels into account—the effects of different air pollutants on the various IR indices are not consistent. Interestingly, exposure to the six air pollutants was not significantly associated with the TyG index, but there was some association with both the TyG-BMI and TyG-WC. This may suggest that air pollution exposure primarily influences IR through its effect on obesity-related indicators. Our mediation analysis results also partially support this notion. Besides, some of the self-report questionnaire results might be subject to recall bias. We did not consider the short-term effects of air pollution exposure. Given the conclusions and limitations of this study, future research should consider employing randomized controlled trial designs to better simulate the impact of air pollution exposure. Nevertheless, our study provides valuable insights and serves as a foundation for future investigations.

## Conclusion

In conclusion, our study obtained new evidence on the association between air pollution exposure and IR in a large cohort of Chinese middle-aged and older individuals. Both individual pollutants and their combined exposure significantly influenced IR. These findings underscore the need for policy interventions aimed at reducing air pollution, particularly in urban areas, to protect vulnerable populations from the detrimental health effects of pollutant exposure. Further research is needed to explore the biological mechanisms underlying these associations and to develop effective strategies for mitigating the health risks posed by expose to air pollution.

## Data Availability

The raw data supporting the conclusions of this article will be made available by the authors, without undue reservation.

## Footnotes

^1^ https://www.geodata.cn

## Glossary

CHARLS: China Health and Retirement Longitudinal Study
CHAP: China High Air Pollutants
PM: Particulate matter
SO_2_: Sulfur dioxide
NO_2_: Nitrogen dioxide
CO: Cobalt
O_3_: Ozone
NO_x_: Nitrogen oxides
AQI: Air quality index
TyG: Triglyceride-Glucose
TyG-BMI: Triglyceride–glucose–body mass index
TyG-WC: Triglyceride–glucose–waist circumference
METS-IR: Metabolic score for insulin resistance
CRP: C-reactive protein
BUN: Blood urea nitrogen
BKMR: Bayesian kernel machine regression
WQS: Weighted quantile sum
Qgcomp: Quantile-based g computation
BMI: Body mass index
WC: Waist circumference
IR: Insulin resistance
IQR: Interquartile range
