# Supplementary material for: Relationship between air pollution exposure and insulin resistance in Chinese middle-aged and older populations: evidence from Chinese cohort
Source: Front Public Health. 2025 Apr 2;13:1551851. doi: 10.3389/fpubh.2025.1551851 (PMC12000001; doi:10.3389/fpubh.2025.1551851)
Supplement: Supplementary file 1 [file Table_1.docx]

**Supplementary figure 1：Flowchart**

**
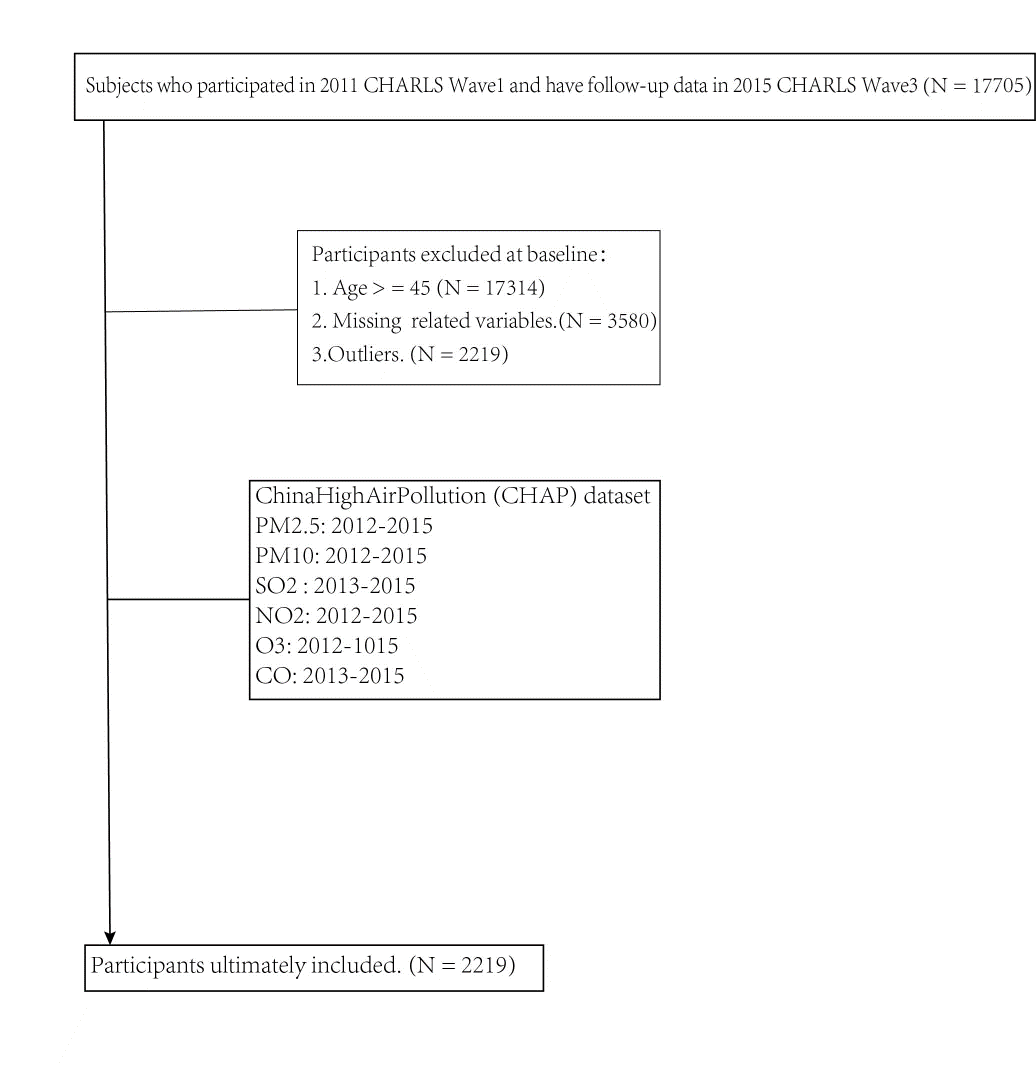
**

**Supplementary table 1: Daily activity scores**

|  | | | | | | |  |
| --- | --- | --- | --- | --- | --- | --- | --- |
| **Question protocol** | | | | | | | **Score** |
| **1. Flat ground activities** | | | | | | |  |
| Have no difficulty with running or jogging about 1 Km | | | | | | | 3 |
| Have no difficulty with walking 1 km | | | | | | | 2 |
| Have no difficulty with walking 100 metres | | | | | | | 1 |
| None of the above activities could be completed independently | | | | | | | 0 |
| **2. Do you have difficulty with getting up from a chair after sitting for a long period?** | | | | | | |  |
| No, I don’t have any difficulty | | | | | | | 3 |
| I have difficulty but can still do it | | | | | | | 2 |
| Yes, I have difficulty and need help | | | | | | | 1 |
| I can not do it | | | | | | | 0 |
| **3. Do you have difficulty with climbing several flights of stairs without resting?** | | | | | | |  |
| No, I don’t have any difficulty | | | | | | | 3 |
| I have difficulty but can still do it | | | | | | | 2 |
| Yes, I have difficulty and need help | | | | | | | 1 |
| I can not do it | | | | | | | 0 |
| **4. Do you have difficulty with stooping, kneeling, or crouching?** | | | | | | |  |
| No, I don’t have any difficulty | | | | | | | 3 |
| I have difficulty but can still do it | | | | | | | 2 |
| Yes, I have difficulty and need help | | | | | | | 1 |
| I can not do it | | | | | | | 0 |
| **5. Do you have difficulty with reaching or extending your arms above shoulder level?  (he/she is regarded as not having difficulty only if he/she can extend both of his/her  arms, otherwise he/she is regarded as having difficulty.)** | | | | | | |  |
| No, I don’t have any difficulty | | | | | | | 3 |
| I have difficulty but can still do it | | | | | | | 2 |
| Yes, I have difficulty and need help | | | | | | | 1 |
| I can not do it | | | | | | | 0 |
| **6. Do you have difficulty with lifting or carrying weights over 10 jin(5 kg), like a heavy bag  of groceries?** | | | | | | |  |
| No, I don’t have any difficulty | | | | | | | 3 |
| I have difficulty but can still do it | | | | | | | 2 |
| Yes, I have difficulty and need help | | | | | | | 1 |
| I can not do it | | | | | | | 0 |
| **7. Do you have difficulty with picking up a small coin from a table?** | | | | | | |  |
| No, I don’t have any difficulty | | | | | | | 3 |
| I have difficulty but can still do it | | | | | | | 2 |
| Yes, I have difficulty and need help | | | | | | | 1 |
| I can not do it | | | | | | | 0 |
|  | | | | | | |  |
| **Supplementary table 2：The association between air pollution and BMI** | | | | | | |  |
|  | | **BMI(β-Coefficients(95%CI))** | | |  |  |  |
|  | | Adjusted Model | | |  |  |  |
| PM_2.5_ | | 0.031(0.024, 0.037)*** | | |  |  |  |
| PM_10_ | | 0.018(0.014, 0.022)*** | | |  |  |  |
| SO_2_ | | 0.045(0.034, 0.056)*** | | |  |  |  |
| NO_2_ | | 0.066(0.053, 0.080)*** | | |  |  |  |
| CO | | 0.007(0.004, 0.010)*** | | |  |  |  |
| O_3_ | | 0.047(0.027, 0.066)*** | | |  |  |  |
| Adjusted Model: Age, gender, marital status, insurance coverage, education, residence, cooking fuel, nighttime, daily activity, alcohol consumption, smoking status, hypertension, diabetes, dyslipidemia, CRP, BUN, air humidity, average temperature  **Supplementary table 3：The association between mixtures and IR indices based on qgcomp model** | | | | | | |  |
| **Air pollutions** | **METS-IR** | | **TyG-BMI** | **TyG-WC** | |  |  |
| Estimate(95%CI) | 1.526(1.035, 2.016) | | 7.612(5.040, 10.185) | 25.381(17.345, 33.417) | |  |  |
| Standard error | 0.25 | | 1.31 | 4.10 | |  |  |
| P-value | 1.25E-09 | | 7.61E-09 | 7.12E-10 | |  |  |

**Supplementary table 4：Primary results after excluding participants diagnosed with malignant tumor(By FDR)**

|  | **TyG (β-Coefficients(95%CI))** | |
| --- | --- | --- |
|  | Crude Model | Adjusted Model |
| PM_2.5_ | 0.001(-0.001, 0.001) | -0.001(-0.001, 0.001) |
| PM_10_ | 0.000(-0.001, 0.001) | -0.001(-0.001, 0.001) |
| SO_2_ | 0.001(-0.001, 0.002) | -0.002(-0.004, 0.001) |
| NO_2_ | 0.002(-0.001, 0.004) | -0.001(-0.002, 0.002) |
| CO | 0.000(-0.000, 0.000) | 0.000(-0.001, 0.000) |
| O_3_ | 0.005(0.001, 0.008)* | 0.003(-0.001, 0.006) |
|  | **TyG-BMI (β-Coefficients(95%CI))** | |
|  | Crude Model | Adjusted Model |
| PM_2.5_ | 0.330(0.252, 0.408)*** | 0.044(-0.002, 0.090) |
| PM_10_ | 0.192(0.148, 0.235)*** | 0.027(0.001, 0.053) |
| SO_2_ | 0.460(0.354, 0.545)*** | 0.087(0.025, 0.152)* |
| NO_2_ | 0.677(0.524, 0.830)*** | 0.138(0.048, 0.227)** |
| CO | 0.009(0.006, 0.013)*** | 0.003(0.001, 0.005)** |
| O_3_ | 0.648(0.425, 0.871)*** | 0.161(0.032, 0.290)* |
|  | **TyG-WC (β-Coefficients(95%CI))** | |
|  | Crude Model | Adjusted Model |
| PM_2.5_ | 1.147(0.901, 1.392)*** | 0.260(0.084, 0.436)** |
| PM_10_ | 0.674(0.539, 0.810)*** | 0.153(0.042, 0.262)* |
| SO_2_ | 1.480(1.149, 1.811)*** | 0.326(0.041, 0.611)* |
| NO_2_ | 2.309(1.829, 2.788)*** | 0.677(0.324, 1.030)*** |
| CO | 0.033(0.022, 0.043)*** | 0.001(0.000, 0.001) |
| O_3_ | 2.476(1.775, 3.177)*** | 0.680(0.158, 1.202)* |
|  | **METS-IR (β-Coefficients(95%CI))** | |
|  | Crude Model | Adjusted Model |
| PM_2.5_ | 0.064(0.050, 0.078)*** | 0.011(0.003, 0.020)* |
| PM_10_ | 0.038(0.030, 0.046)*** | 0.006(0.001, 0.012)* |
| SO_2_ | 0.092(0.073, 0.111)*** | 0.018(0.003, 0.032)* |
| NO_2_ | 0.134(0.106, 0.162)*** | 0.003(0.012, 0.048)** |
| CO | 0.001(0.001, 0.002)*** | 0.001(0.000, 0.001)** |
| O_3_ | 0.139(0.100, 0.180)*** | 0.023(-0.003, 0.050) |

Adjusted Model: Age, gender, marital status, insurance coverage, education, residence, cooking fuel, nighttime, daily activity, alcohol consumption, smoking status, hypertension, diabetes, dyslipidemia, CRP, BUN, TyG/TyG-BMI/TyG-WC/METS-IR in 2011, air humidity, average temperature

**Supplementary table 5：Primary results after excluding participants receiving antidiabetic medications or insulin treatment(By FDR)**

|  | **TyG (β-Coefficients(95%CI))** | |
| --- | --- | --- |
|  | Crude Model | Adjusted Model |
| PM_2.5_ | 0.000(-0.001, 0.002) | -0.001(-0.002, 0.001) |
| PM_10_ | 0,000(-0.000, 0.000) | -0.001(-0.001, 0.000) |
| SO_2_ | 0.000(-0.001, 0.002) | -0.002(-0.004, 0.001) |
| NO_2_ | 0.001(-0.002, 0.004) | 0.000(-0.002, 0.002) |
| CO | 0.000(-0.000, 0.000) | 0.000(-0.000, 0.000) |
| O_3_ | 0.003(-0.001, 0.007) | 0.002(-0.001, 0.005) |
|  | **TyG-BMI (β-Coefficients(95%CI))** | |
|  | Crude Model | Adjusted Model |
| PM_2.5_ | 0.320(0.241, 0.399)*** | 0.046(-0.001, 0.009) |
| PM_10_ | 0.189(0.144, 0.233)*** | 0.031(0.004, 0.058)* |
| SO_2_ | 0.452(0.345, 0.558)*** | 0.088(0.023, 0.152)* |
| NO_2_ | 0.650(0.495, 0.805)*** | 0.133(0.041, 0.224)** |
| CO | 0.009(0.006, 0.013)*** | 0.003(0.001, 0.004)** |
| O_3_ | 0.587(0.360, 0.814)*** | 0.169(0.037, 0.300)* |
|  | **TyG-WC (β-Coefficients(95%CI))** | |
|  | Crude Model | Adjusted Model |
| PM_2.5_ | 1.098(0.851, 1.345)*** | 0.272(0.093, 0.451)** |
| PM_10_ | 0.639(0.501, 0.777)*** | 0.162(0.049, 0.275)** |
| SO_2_ | 1.436(1.102, 1.769)*** | 0.334(0.042, 0.627)* |
| NO_2_ | 2.215(1.730, 2.700)*** | 0.675(0.315, 1.036)*** |
| CO | 0.032(0.021, 0.042)*** | 0.009(0.001, 0.017) |
| O_3_ | 2.250(1.539, 2.961)*** | 0.705(0.171, 1.238)* |
|  | **METS-IR (β-Coefficients(95%CI))** | |
|  | Crude Model | Adjusted Model |
| PM_2.5_ | 0.062(0.048, 0.076)*** | 0.012(0.003, 0.020)* |
| PM_10_ | 0.037(0.029, 0.045)*** | 0.007(0.001, 0.012)* |
| SO_2_ | 0.091(0.071, 0.110)*** | 0.017(0.002, 0.031)* |
| NO_2_ | 0.130(0.102, 0.158)*** | 0.028(0.010, 0.047)** |
| CO | 0.002(0.001, 0.002)*** | 0.000(0.000, 0.001)** |
| O_3_ | 0.129(0.088, 0.170)*** | 0.023(-0.004, 0.050) |

Adjusted Model: Age, gender, marital status, insurance coverage, education, residence, cooking fuel, nighttime, daily activity, alcohol consumption, smoking status, hypertension, diabetes, dyslipidemia, CRP, BUN, TyG/TyG-BMI/TyG-WC/METS-IR in 2011, air humidity, average temperature
